# Aldosterone and Mineralocorticoid Receptors—Physiology and Pathophysiology

**DOI:** 10.3390/ijms18051032

**Published:** 2017-05-11

**Authors:** John W. Funder

**Affiliations:** 1 Hudson Institute of Medical Research, 27–31 Wright St, Clayton 3168, Victoria, Australia; john.funder@hudson.org.au; 2 Monash University, Clayton 3800, Victoria, Australia

**Keywords:** endogenous ouabain, cortisol, sodium and primary aldosteronism

## Abstract

Aldosterone is a uniquely terrestrial hormone, first appearing in lungfish, which have both gills and lungs. Mineralocorticoid receptors (MRs), on the other hand, evolved much earlier, and are found in cartilaginous and bony fish, presumptive ligand cortisol. MRs have equivalent high affinity for aldosterone, progesterone, and cortisol; in epithelia, despite much higher cortisol circulating levels, aldosterone selectively activates MRs by co-expression of the enzyme 11β-hydroxysteroid dehydrogenase, Type 11. In tissues in which the enzyme is not expressed, MRs are overwhelmingly occupied but not activated by cortisol, which normally thus acts as an MR antagonist; in tissue damage, however, cortisol mimics aldosterone and acts as an MR agonist. The risk profile for primary aldosteronism (PA) is much higher than that in age-, sex-, and blood pressure-matched essential hypertensives. High levels of aldosterone per se are not the problem: in chronic sodium deficiency, as seen in the monsoon season in the highlands of New Guinea, plasma aldosterone levels are extraordinarily high, but cause neither hypertension nor cardiovascular damage. Such damage occurs when aldosterone levels are out of the normal feedback control, and are inappropriately elevated for the salt status of the individual (or experimental animal). The question thus remains of how excess salt can synergize with elevated aldosterone levels to produce deleterious cardiovascular effects. One possible mechanism is through the agency of the elusive ouabain-like factors (OLFs). Such factors are secreted from the adrenal in response to ACTH (adrenalocortical tropic hormone), to angiotensin via AT2R, and—the polar opposite of aldosterone—to sodium loading. They act on blood vessels to cause vasoconstriction and thus elevate blood pressure to dump excess sodium through pressure natriuresis. Their levels are chronically elevated in PA in response to the continually elevated sodium status, and they thus act to constrict coronary and systemic arteries. In the context of the elevated blood volume and total body sodium in a PA patient, this raises blood pressure and acts as the proximate cause of cardiovascular damage. If this is the case, it would appear to offer new insights into therapy for PA. One would be the use of digibindin, or its more recent successors as antagonists of OLFs acting on Na/K ATPase at the vessel wall. A second would be to routinely combine a low dose MR antagonist, an ENaC inhibitor, and sodium restriction as first-line therapy for bilateral aldosterone overproduction. Finally, for unilateral cases post-surgery, there is good reason to include low-dose MRs in drug therapy if required, given the ability of cortisol in damaged blood vessels to mimic aldosterone vasoconstrictor action.

## 1. Physiology

Commonly, the physiology of aldosterone and mineralocorticoid receptors (MRs) begins (and sometimes, unfortunately ends) with the action of aldosterone via renal MRs to retain sodium (as well as water) and to excrete potassium. This is not surprising, given that aldosterone is characterized by the demonstration of its effects on transepithelial electrolyte transport and what was initially termed “electrocortin” [1]. Similarly, the first identification of high affinity receptors for aldosterone occurred in vitro studies on rat kidney slices [2]. Again, these were initially termed “Type 1 corticosteroid receptors”, in contrast with the dexamethasone-binding Type 2 receptors; now Type 1 and Type 2 are known as MRs and glucocorticoid receptors (GRs). Accordingly, for a considerably nephrological audience, the focus of the current section will be on lesser-known aspects of aldosterone and MR physiology.

**Figure 1:**
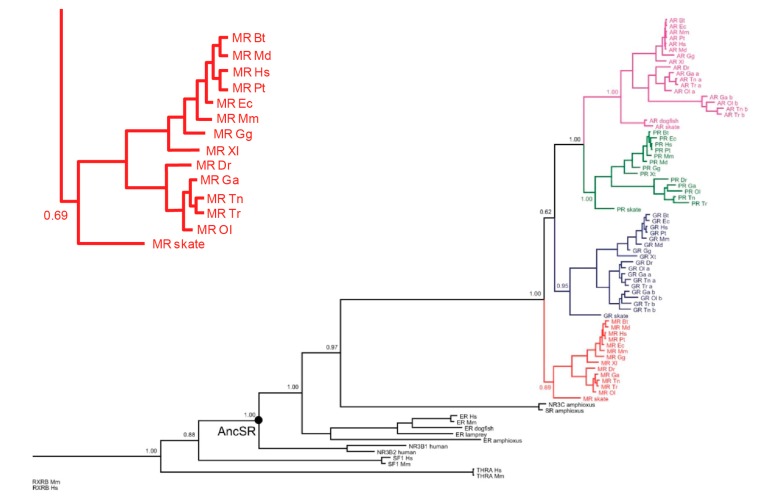
Dendrogram of evolution of mineralocorticoid receptors (MRs). Glucocorticoid receptors (GRs), progesterone receptors (PRs), and androgen receptors (ARs) from common ancestral protein. Inset: MRs across different species expanded. Redrawn from Kassahn et al., 2011 [3].

In all ligand–receptor systems, an obvious question is which came first, signal or receptor—chicken or egg. For aldosterone and MRs, the answer is unequivocal: the MRs preceded aldosterone by millions of years. Figure 1 shows a dendrogram of the evolution of the tight-knit MRs/GRs/progesterone receptors (PRs)/androgen receptors (ARs) subsequently from a common ancestor. The first of the four to branch off is the primordial MRs, as shown in Figure 1; the inset details its presence in a number of species, including bony fish and cartilaginous species such as sharks and rays [3].

**Figure 2:**
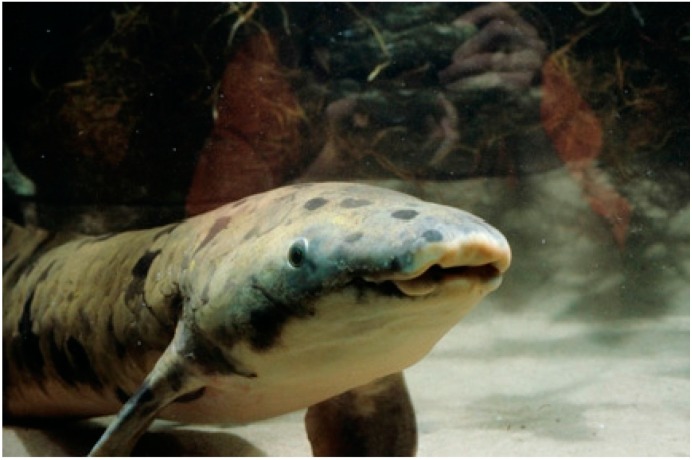
The lungfish, first creature to make aldosterone.

In contrast, Figure 2 shows the first creature in which aldosterone appears, the lungfish. These animals, as their name implies, have both gills and lungs, marking the transition from an obligate aquatic to a terrestrial milieu. The first thing that one needs to know about mineralocorticoid receptors is that they evolved millions of years before aldosterone did, that their early physiologic roles are not yet well-defined, that their probable but not certain ligand was cortisol, and that we avoid these insights on evolution at our peril.

The second thing one needs to know is that MRs are truly promiscuous. We all know that they bind aldosterone with high affinity: in fact, they have equivalent high affinity for a range of steroids—aldosterone, cortisol, corticosterone, deoxycorticosterone (DOC), and progesterone. Given the emergence of aldosterone as the physiologic mineralocorticoid, our terrestrial MRs might have undergone mutation so that they no longer recognized cortisol. This did not happen over the eons, and we must thus face why contemporary MRs remain promiscuous and what this means for physiology and pathophysiology.

For some years before characterization of aldosterone DOC was the ‘gold standard’ mineralocorticoid; in terms of physiology, it appears to be of no real importance in this role. Under normal circumstances its plasma levels are low, about the same as those of aldosterone, but the secretion of DOC, unlike that of aldosterone, is not in response to sodium deficiency or volume depletion [4]. In addition, although DOC and aldosterone have similar high affinity for MRs [5], whereas aldosterone circulates ~50% bound to plasma protein, DOC is 94–99% protein-bound: in vivo it has approximately one-tenth to one-fortieth the mineralocorticoid potency of aldosterone on a dose basis [6]. Pathophysiologically, however, it may come into play in the ectopic secretion of ACTH from malignancies, where the normal feedback loop between adrenal and anterior pituitary is no longer operant.

Corticosterone is the physiologic glucocorticoid in rats and most strains of mice, where its roles are (presumably) parallel those of cortisol in humans. Particular roles for circulating corticosterone distinct from those of cortisol in humans have been suggested but are yet to be proven. Progesterone is also highly protein bound, but in pregnancy circulates at concentrations sufficient to cause a natriuresis, elevating levels of aldosterone 3–10-fold in response. Much lesser elevations of circulating progesterone in the luteal phase of the menstrual cycle have been shown to raise plasma aldosterone concentrations in some women to false-positive aldosterone/renin ratios for primary aldosteronism [7].

Whether the progesterone of pregnancy has a physiologic role as an MR antagonist has not been explored. It may be that its MR antagonist action, the aldosterone elevation in response, and the consequent reset of fluid and electrolyte homeostasis, is nothing more than the biologic equivalent of collateral damage. It may also, however, speak to an as yet unrecognized physiologic role, again given the maintained high affinity of MRs for progesterone over evolution. What has been reported is a single nucleotide mutation in the gene encoding the human MR; the mutant MR sees progesterone as an MR agonist, resulting in early onset hypertension very much exacerbated by pregnancy [8].

Which brings us to cortisol, circulating at plasma total levels ~1000-fold those of aldosterone, and plasma levels “only” ~100-fold higher, given the much higher levels of cortisol binding (~95%) to plasma protein. The remarkable thing about cortisol in its relationship with MRs is that it is bivalent. Normally, it is antagonist, in that it binds but does not activate MRs in the same way as aldosterone does; in the context, however, of tissue damage, reactive oxygen species generation and redox change cortisol becomes an MR agonist, mimicking aldosterone, as detailed below.

In epithelia, and in a few non-epithelial tissues (vessel wall, nucleus tractus solitarius), MRs are protected from activation by cortisol via the twin actions of the enzyme 11βhydroxysteroid dehydrogenase (11βHSD2). In addition to converting cortisol to receptor-inactive cortisone, for every molecule of cortisone so produced, a molecule of NAD is reduced to NADH. Metabolism of cortisol debulks aldosterone target tissues by a factor of ~10, still a ~10-fold excess of cortisol over aldosterone, such that the majority of MRs are occupied but not activated by cortisol. What appears to hold cortisol-MR complexes inactive is in high levels of NADH generated, as previously reported for the corepressor c-terminal-binding protein (CtBP) [9]. When 11βHSD2 is deficient or blocked, both of these ‘protective’ mechanisms fail, and cortisol activates principal cell MRs. In tubular intercalated cells, MRs but not 11βHSD2 is expressed, and the MRs ‘protected’ by phosphorylation at serine^843^ [10]. When, however, they are dephosphorylated in response to angiotensin, they are activated (experimentally) by aldosterone or cortisol—and, physiologically speaking, presumably by cortisol given its orders of magnitude higher levels [11].

**Figure 3:**
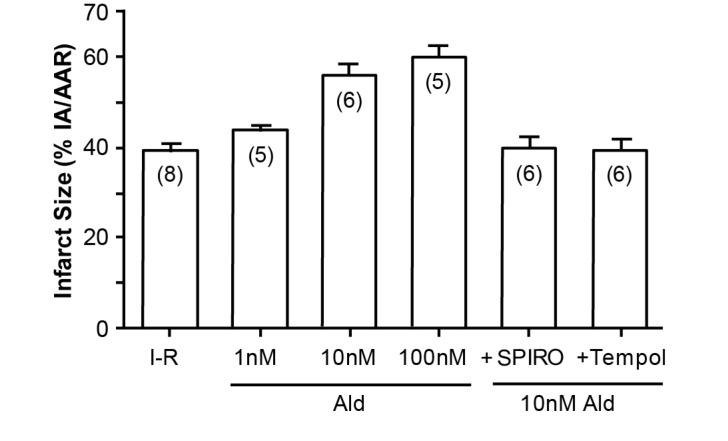
Aldosterone increases infarct size in Langendorf ischemia-reperfusion rat heart preparations. *n* values in parentheses; Ald: aldosterone; SPIRO: spironolactone; from Mihailidou et al., 2009 [13].

**Figure 4:**
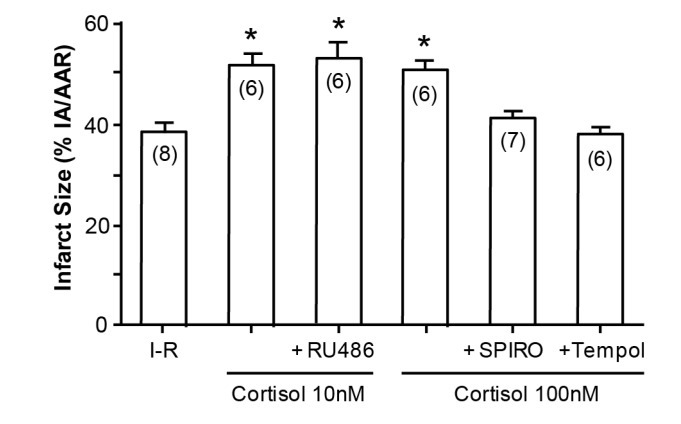
Cortisol increases infarct size in Langendorf ischemia-reperfusion rat heart preparations. * *p* < 0.05, *n* values in parentheses; SPIRO spironolactone; from Mihailidou et al., 2009 [13].

An illustration of how cortisol mimics aldosterone is the study by Mihailidou et al, exploiting the Langendorf rat heart model of experimental ischemia followed by reperfusion. Cardiomyocytes express MRs but not 11βHSD2, so the possibility of a physiologic role for aldosterone is remote. Experimentally, as shown in Figure 3, aldosterone aggravates ischemia–reperfusion induced infarct size, as has been previously reported [12]. What is novel, however, is that cortisol at low doses similarly aggravates tissue damage (Figure 4); that this action is via MRs and not GRs is attested to not merely by the dose, in that 10 and 100 nM are equipotent, but also by the ability of spironolactone—not the GR or the PR antagonist RU486, to reverse the effect of cortisol [13].

Finally, one of the enigmas of the RALES study [14] showing the remarkable effects of spironolactone added to standard of care on mortality and morbidity in congestive heart failure was the very modest average dose of spironolactone (26 mg/day) used. The effects were dramatic (a 30% reduction in mortality, leading to the trial being stopped halfway through), and 35% fewer hospitalizations. Plasma aldosterone concentrations were in the low–normal range, and the unprotected MRs were activated by normal levels of cortisol: how is it, then, that low-dose spironolactone proved so efficacious?

**Figure 5:**
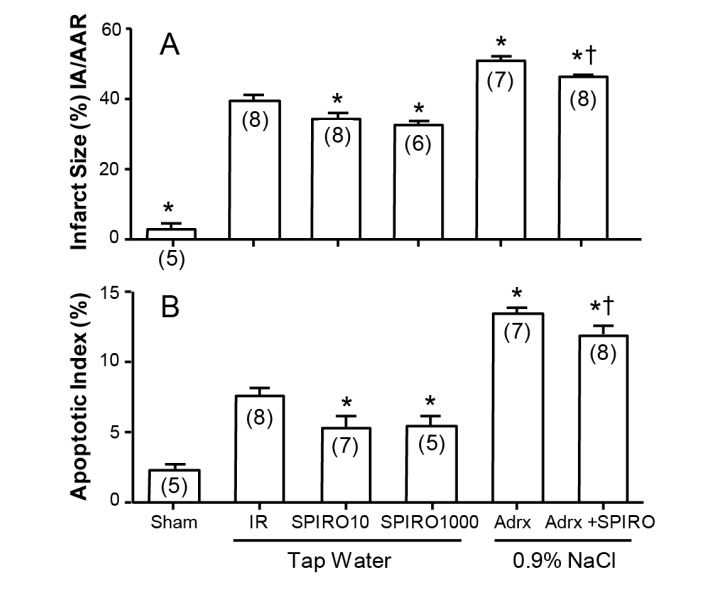
Spironolactone lowers infarct size (**A**) and apoptotic index (**B**) in Langendorf ischemia-reperfusion rat heart preparations, in both adrenal intact rats (Columns 3 & 4) and adrenalectomized rats (Columns 5 & 6) * significantlydifferent from IR baseline ischemia-reperfusion, † significantly different from adrx alone; from Mihailidou et al., 2009 [13].

A clue to the answer may lie in Figure 5, where the effects of spironolactone absent any other steroid in infarct size and area at risk are shown in the Langendorf model [10]. Whether the Langendorf preparation was from intact rats maintained on tap water, or from rats adrenalectomized one week previously and maintained on 0.9% NaCl to drink, spironolactone reduced both infarct area (Figure 5A) and apoptotic index (Figure 5B). The rats adrenalectomized (to exclude any residual corticosteroid) are understandably more fragile than intact rats, but still show significantly lower levels with spironolactone: in both cases, the effect is due to spironolactone acting as an inverse agonist to reduce cell death at the infarct margin. What this means is that spironolactone does not act merely as a “blocker”, as there is no corticosteroid to block, but acts putatively as an intracellular antagonist of MR activation beyond the receptor.

## 2. Pathophysiology

Over a decade ago, two studies reported patients with primary aldosteronism as having a higher cardiovascular risk profile [15] or with evidence of cardiovascular damage [16] than age-, sex- and blood pressure-matched essential hypertensives. The first was from Parisian inpatients with long-standing blood pressure elevation; those with primary aldosteronism had a 4.2 times higher risk of stroke, 6.5 times the risk of non-fatal myocardial infarct, and 12.1 times the prevalence of atrial fibrillation [15].

The second study, published at the same time, addresses the effects of very early hyperaldosteronism [16]. Michael Stowasser and his colleagues compared eight young, normotensive patients with proven familial hyperaldosteronism, Type 1, case-matched with 24 normotensive controls. Those with primary aldosteronism had a thicker left ventricular wall and reduced diastolic function compared with the controls, evidence that inappropriate aldosterone levels are deleterious, even in the absence of elevated blood pressure.

The keyword here is inappropriate. In the highlands of New Guinea, yams are the staple food, and in the monsoon season, the rains leach the minerals from the soil. As a result, the average daily intake of sodium is very low, and excretion commonly 2–3 meq/day. These subjects have low normal blood pressure, no cardiovascular damage, and extraordinarily high plasma aldosterone concentrations. What this means is that physiologically circulating aldosterone levels can be very high, in homeostatic mode responding to sodium deficiency, without the deleterious effects seen in primary aldosterone patients with much lower levels of plasma aldosterone.

Here, the keyword again is appropriate. When very high aldosterone levels are appropriate for the degree of sodium deficiency, they are homeostatic—“a good thing”. When aldosterone levels are out of the normal feedback control loop, and inappropriate for sodium status, cardiovascular damage ensues—“a bad thing”. Currently, our clinical focus is on aldosterone as the culprit, with posited direct effects on the heart and on blood vessels, producing early and late indices of cardiovascular damage detailed above [12,13]. What has been neglected is the obvious crucial role of salt—and the fact that, without salt, even sky-high aldosterone levels are benign.

One possible mechanism to explain this apparent dilemma is the agency of the elusive endogenous ouabain (EO), or ouabain-like compounds (e.g., marinobufagenin), hereafter referred to as EO. Secretion of these compounds is from the adrenal cortex at very low levels, similar to those of aldosterone. ACTH increases the secretion of EO, and ACTH-driven experimental hypertension is reversed by the administration of digibindin [17], as its name implies an EO blocker. Aldosterone is similarly elevated by ACTH, to which it is particularly sensitive in ~25% of hypertensives, elevating aldosterone at doses too low to increase cortisol [18].

The two diverge to a degree in terms of their responsiveness to angiotensin. Angiotensin II stimulates secretion of both, but crucially through different receptors—AT_1_R for aldosterone, AT_2_R for EO [19], an important difference given the order of magnitude of lower affinity of the AT_2_R. The final divergence is total, and crucial to the presumed physiologic rule of EO, and its possible role in primary aldosteronism, EO secretion is increased by sodium loading, whereas that of aldosterone is decreased, a polar opposite effect [20].

Like exogenous ouabain, EO inhibits the α_2_ subunit of the Na^+^/K^+^ pump. In kidney tubules, this reduces sodium reabsorption; in vascular smooth muscle cells, it induces Na^+^ and Ca^2+^ accumulation, vasoconstriction and systemic hypertension [21]. In terms of physiology then, its role would appear to be to increase natriuresis, pressure-induced via vasoconstriction, directly at the level of the renal tubule, in a homeostatic response to sodium loading. Pathophysiologically, however, when aldosterone is out of its normal homeostatic feedback control—which is the case in primary aldosteronism—the inappropriate sodium retention indicates the continued elevation of EO in an attempt at natriuresis.

In addition to seeking direct effects of aldosterone on blood vessels—for which there is clear evidence [22,23]—and on the cardiomyocyte—for which (given unprotected MRs) there is not—we thus need to factor in the lessons from New Guinea. When aldosterone levels are elevated in sodium deficiency, they serve to constrict blood vessels to offset, at least in part, the concomitant volume contraction: homeostasis again [24]. Under such circumstances, the secretion of EO is attenuated, and aldosterone does no cardiovascular damage.

If this is the case, in part or in full, there are a number of things that flow in terms of the ultimate treatment of PA. Young female patients in whom an aldosterone-producing adenoma (APA) is detected by prompt screening and lateralization have the best chance of completing clinical remission post-operatively. In males and with age, the percentage of APA patients cured by unilateral adrenalectomy falls, so that in a yet unpublished study, the average cure rate was 37% across 12 expert centers using the same criteria for complete clinical remission.

Across a range of studies, a biochemical cure—i.e., plasma [K^+^] and plasma aldosterone concentration (PAC) returning to normal levels—is, commonly almost 100%. In some patients, clinical remission is partial—blood pressure is lower but is still elevated and necessitates medication. In some patients, remission is missing, with no appreciable fall in blood pressure or in the defined daily dose of medications. In the former, which is the larger group, persistent hypertension is attributed to pre-existing vascular damage, eminently feasible given the common long period between the onset of hypertension and surgical intervention. In the group with no clinical remission, age clearly plays a role; in subjects with imperfect biochemical remission, the possibility of bilateral nodules unsuspected on imaging cannot be ruled out.

The other group, which is much larger than that of patients with APA, is that of patients with bilateral adrenal hyperplasia (BAH). In these patients, primary aldosteronism is commonly less florid, so that, in centers using very straitened cutoffs, the ratio of APA-to-BAH is close to 1; in centers using more relaxed cut-offs, the ratio of verified primary aldosteronism is 1:2 for APA/BAH. Currently, there is mounting evidence that inappropriate aldosterone secretion—also known as primary aldosteronism—may have an overall prevalence of up to 50% in hypertensives [18,25], and of the order of 15–20% in normotensives [26,27].

The current treatment for BAH is relatively low-dose mineralocorticoid receptor antagonists (MRAs)—12.5–50 mg/day for spironolactone and 50–100 mg/day for eplerenone, with additional agents if and as required to bring blood pressure into the normal range. Neither of the current MRAs is optimal: in men, spironolactone has side effects (gynecomastia and erectile dysfunction), mirroring its AR antagonist activity; in women, effects via activating PRs in the menstrual cycle, and mastodynia. Eplerenone is expensive, and in some jurisdictions reimbursable only for heart failure. Although the percentage of disabling side effects of low-dose spironolactone is not high, it is not popular with patients, and adherence is often a problem.

Over the next five years, it is to be hoped that therapy for incomplete remission following APA/BAH is refined in three ways, with the possible emergence of a fourth. First is to recognize that salt is the driver, and that patients in both APA post-surgery and BAH limit their salt intake. The second is the development of potent, selective, non-steroidal MRAs, widely available and recompensable, for patients with BAH. The third is the recognition that, despite their normal aldosterone levels, APA patients with incomplete or missing clinical remission still have cortisol activating their vascular smooth muscle MR—and an MRA should be the first-line hypertensive in such patients, with conventional agents to follow if necessary.

The reason that this is not currently the case is ignorance, plus a misnomer. The misnomer is the persisting “aldosterone receptor”: if you have fixed the aldosterone, why pay attention to the receptor? The ignorance is of the evolution, physiology, and pathophysiology of the MRs, even among those who are otherwise experts in managing primary aldosteronism. It is increasingly recognized that MRAs should be used in resistant hypertension and low-renin hypertension (and that in these categories 15–70% of patients may in fact have primary aldosteronism); there are compelling data for eplerenone as a potent hypotensive in essential hypertensives with normal plasma renin, aldosterone, and potassium [28]. In studies on DOCA/salt rats, blood pressure and indices of cardiovascular damage progressively increased [29,30]. Those given DOCA for the first four weeks, but in which salt intake continued until the eighth week, showed blood pressure and fibrosis levels equivalent to those of DOCA/salt rats killed at four weeks. Similarly fed rats (4 weeks DOCA, 8 weeks salt) given eplerenone from weeks 5–8 returned to a normal blood pressure with a resolution of cardiovascular damage.

Unless a patient is cured by surgery—and this is likely to be a very small percentage, as inappropriate aldosterone secretion is increasingly recognized as primary aldosteronism—all other patients need to receive low-dose MRAs as a first-line antihypertensive. Now, MRA therapy is spironolactone, eplerenone if affordable, and canrenone where available. They also need to halve (probably) their salt intake at the same time. When Generation 3 MRAs (which are non-steroidal, cheap to produce, modestly priced, as potent as spironolactone, and as selective as eplerenone) emerge, they can jettison their existing MRAs. Importantly, Generation 4 MRAs (all the above plus tubule-sparing), the current focus of much of the development, are specifically contra-indicated for this growing group of subjects.

The fourth and as yet final possible therapeutic option is that of EO blockers/antagonists. Over 20 years ago, it was reported that “aldosterone antagonists” (sic) inhibited ouabain-like factors, based on in vivo observations: it is probable that this was a secondary effect, mediated via natriuresis. A decade ago, Rostafuroxin (PST 2238) was shown to be a safe and very effective antagonist both of EO- and α-adducin induced hypertension [31]. In more recent animal studies [32], Rostafuroxin has been shown to be renoprotective, blunting EO-induced changes in renal ischemia-induced injury. Whether or not EO antagonists might have a place in the management of PA is yet to be explored.
